# Factors Influencing Quality of Life after Massive Weight Loss—What Makes the Difference?

**DOI:** 10.3390/healthcare11081147

**Published:** 2023-04-17

**Authors:** Matthias Michael Aitzetmüller-Klietz, Laura Raschke, Tobias Hirsch, Maximilian Kückelhaus, Philipp Wiebringhaus, Marie-Luise Aitzetmüller-Klietz, Kamran Harati

**Affiliations:** 1 Division for Plastic Surgery, Department of Trauma, Hand and Reconstructive Surgery, University Hospital Muenster, Waldeyerstrasse 1, 48149 Muenster, Germany; 2 Department for Plastic and Reconstructive Surgery, Institute for Muskuloskeletal Medicine, Westfaelian Wilhelms-University Muenster, 48149 Münster, Germany; 3 Plastic, Reconstructive, and Aesthetic Surgery, Hand Surgery, Fachklinik Hornheide, Dorbaumstrasse 300, 48157 Muenster, Germany

**Keywords:** BODY-Q, quality of life, body contouring, massive weight loss

## Abstract

Although weight reduction in obesity and morbid obesity has been shown to improve associated comorbidities, there is currently no information on what influences quality of life after a large reduction in body weight. The present study looks at differences in patients’ quality of life classified by mode and amount of weight loss. Material and methods: A cross-sectional study was designed using a validated German version of the BODY-Q questionnaire. The internet-based questionnaire was distributed to patients via social media. Results: 460 patients (443 female, 17 male) were interviewed for this study via “Surveymonkey”. The comparison of conservative and surgical weight loss showed no significant difference in the patients’ quality of life (*p* > 0.05). A high BMI correlates negatively with body image (*p* = 0.023 *), as does the specific assessment of most body regions. For example, a negative correlation was found between a high BMI and satisfaction with skin appearance (*p* < 0.001 *) and satisfaction with the inner thigh (*p* = 0.011 *). Conclusion: Increased weight loss is associated with a greater ability to maximise quality of life. The type of weight loss, whether conservative or surgical, can be neglected based on the present study. Bariatric surgery cannot be considered a universal solution to obesity. Body contouring interventions should also become a focus of therapy.

## 1. Introduction

The global increase in obesity, with a global prevalence of 39% [1] represents a tremendous global health problem [2]. Obesity and its comorbidities are thus increasingly becoming a challenge with significant economic and medical implications [3]. The German population shows a male dominance of obesity prevalence at 62%, and an increase in prevalence with age [4]. An association with an enhanced mortality rate [5], comorbidities such as type 2 diabetes mellitus and cardiovascular disease are common within this group [5]. 

Whether a person is considered obese or overweight is assessed by the body mass index (BMI-kg/m^2^) [6]. This score was created as a risk indicator for diseases. Consequently, high BMI goes along with comorbidities such as hypertension, osteoarthritis, premature death and diabetes [7]. While the official classification according to the World Health Organization (WHO) can be found in Table 1, it should be noted that the BMI should only serve as a guideline and is not representative of a person’s state of health alone [6]. 

From a health–economic perspective, the health status of a population can be assessed on the basis of the disease spectrum “overweight and obesity“ [4]. Despite its recognition as a chronic disease by the WHO in 2000, obesity still lacks social acceptance [8]. In 2016, the German sustainability strategy set the goal of preventing the population share of people with obesity in Germany from growing [9]. Apart from a patient’s physical health status, self-perception and own body image are gaining in importance [4,8]. Success of a medical intervention is no longer judged solely by the therapy or surgical result. Evaluation of the quality-adjusted life years (QALY) or disability-adjusted life years (DALY) represent essential tools for efficiency calculation and cost–benefit optimization in today’s health care system [10,11,12].

Quality of life (QoL) is generally defined as an individual assessment of a person’s well-being with themselves and their environment and even ranked higher than life expectancy [13,14]. If the focus lays on patient-centred health status in addition to the QoL survey, this is referred to as health-related quality of life (HRQoL) [14,15,16,17]—nowadays used as an objectifiable indicator of therapy success [18]. 

In some cases, obesity is reduced by either lifestyle changes or bariatric surgery and thereby followed by massive weight loss (MWL). The influence of obesity on HRQoL has been studied several times indicating that high BMI decreases HRQoL [19,20]. While studies have shown that weight reduction, whether conservative or surgical and regardless of the extent, always improves obesity-associated comorbidities [21], there only exist few analyses of the effect of MWL on QoL. Certain parameters such as patient dependent factors or factors that might interfere with weight loss and might influence HRQoL of these patients are not described at all.

Therefore, the aim of this study is to examine the effect of massive weight loss (MWL) from different psychosocial aspects. Based on our literature review, there are currently no analyses investigating parameters that influence QoL of MWL patients.

## 2. Material and Methods

### 2.1. Study Design

The present study was designed as an anonymized, non-randomized, prospective study based on an official German version of the BODY-Q and distributed via the online platform “Survey Monkey” (Surveymonkey, San Mateo, CA, USA). The link was distributed via patient events (at the Fachklinik Hornheide and at congresses) and seminars and more important via social media. Thereby “Instagram” and “Facebook” (both Meta Platforms, Menlo Park, CA, USA) served as main distribution platforms. Distributing focused on sharing the link to the questionnaire in Facebook groups as well as on the Instagram accounts of the authors. Groups were specifically selected to focus on the following core topics: body contouring procedures, massive weight loss, bariatric surgeries and excess skin. All participants voluntarily completed the survey between 25 November 2020 and 21 January 2021.

### 2.2. Inclusion Criteria

Consideration was given if the input questions were answered completely. Further inclusion was performed separately for each subcategory. Incomplete answering of sections led to exclusion separately for each subgroup. The above individual analysis of the individual subgroups led to a variance in the number of participants between 224 and 460, depending on the category.

### 2.3. Measurement Tools and Software Used

Quality of life was assessed using a linguistically validated official German version of the BODY-Q questionnaire [22]. All 26 scales of the questionnaire: psychosocial distress due to external appearance, expectations, body image, physical function, psychology, sexuality, society, satisfaction with the abdomen (Z Abdomen), body (ZK), upper arms (ZOA), back (ZR), buttocks (Z Buttock), hips and outer thighs (ZHOA), inner thighs (ZOI), assessment of excess skin (Skin), assessment of scars after body contouring surgery, satisfaction with the attending physician, satisfaction with the information, satisfaction with the medical staff and satisfaction with the administrative staff were collected.

Data collection and initial trend analysis were carried out completely anonymously with “Surveymonkey”. Further statistical analysis was performed using Microsoft Excel 2019 (Microsoft Corp., Redmond, WA, USA) and the statistical program “R” (1.4.1103, RStudio: Integrated Development for R., RStudio PBC, Boston, MA, USA). Plots (e.g., boxplots), distributions and tables were created for visual representation.

### 2.4. Data Analysis

Respondents were classified based on the method of weight loss (conservative and surgical—group 1 and group 2). A non-parametric Wilcoxon test was used for comparison. A *p*-value of <0.05 was considered as statistically significant.

Additionally, the effects of the BMI level on body image and appearance in certain areas regarding QoL were calculated. A regression analysis according to Pearson was carried out for BMI and BQ value.

The relative effect (estimator) was set as the effect measure. The confidence interval was set at 95% and a *p*-value < 0.05 (two-sided) was considered as statistically significant. The estimator, upper limit, lower limit and *p*-value of the relative effect analysis were calculated. The groups studied (1 and 2, A and B) were considered independently. The relative effect was defined from 0 (group 1) to 1 (group 2).

Furthermore, the patients were clustered into four subgroups based on the amount of weight loss (groups A–D; Group A: 0 kg to 30 kg; Group B: 30 kg to 50 kg; Group C: 50 kg to 70 kg; Group D > 70 kg). Using the Kruskal–Wallis test, the primary analysis evaluated significances between groups. The post-high analysis was carried out using a paired Wilcoxon test aiming to identify significant differences among the various groups. Hereby, a *p*-value of <0.1 was considered as statistically significant.

Additionally, a regression analysis was performed. In the case of ordinal data, a regression analysis according to Spearman was carried out with calculation of the *p*-value and rho value in selected BODY-Q categories. For the metric variable BMI, the regression analysis according to Pearson was chosen. *p*-value, Pearson correlation coefficient and the adjusted coefficient of determination R are documented.

## 3. Results

### 3.1. Patient Demographics

In total, the online survey was started 471 times. A total of 11 participants were excluded due to repeated participation or incompleteness of initial questions. A total of 460 patients (97.67%—443 women (96.3%) and 17 men (3.7%)) with a mean age of 43 years (±0.73 SD) met our inclusion criteria and were included in the study. Mean current BMI was 29.54 kg/m^2^ (±0.31 SD) with a mean maximum weight of 174.43 kg (±8.31 SD) and a mean current weight of 84.61 kg (±0.92 SD).

### 3.2. Results BODY-Q

Patient satisfaction was assessed on a scale of 0 to 100 points according to a previously published outcome algorithm by Klassen et al. [23].

**Figure 1:**
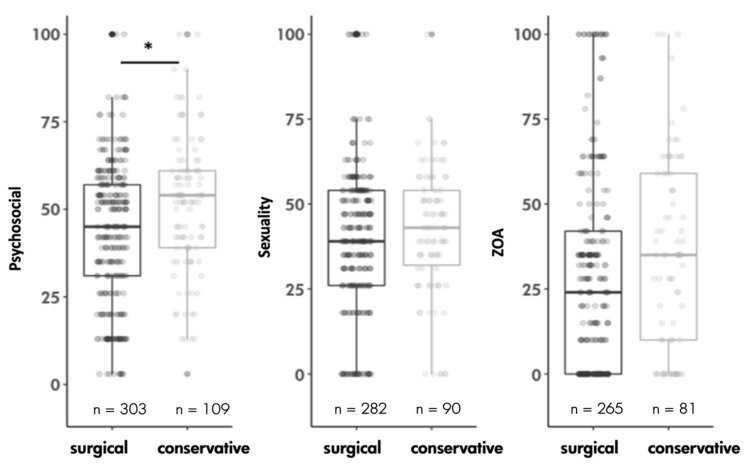
Comparison of the quality of life in the subscales “psychosocial”, “sexuality” and “satisfaction-upper arms” of the two patient groups weight loss by conservative measures vs. weight loss by surgical measures. The box plots represent the median, the interquartile range and the distribution. Statistical differences were examined using the Wilcoxon test. The significance level is * *p* < 0.

Participants were divided according to the form of weight loss. Group 1 (126 patients (27.4%) used conservative measures and group 2 (334 patients (72.6%) underwent bariatric surgery. Comparison showed no significant difference in almost all categories of the BODY-Q, except in the category “psychosocial” with a significantly higher QoL in group 2 (average difference of 8.28 points (*p* = 0.0005 *, estimator: 0.6096, Figure 1)). Detailed values are listed in Table 2.

**Figure 2:**
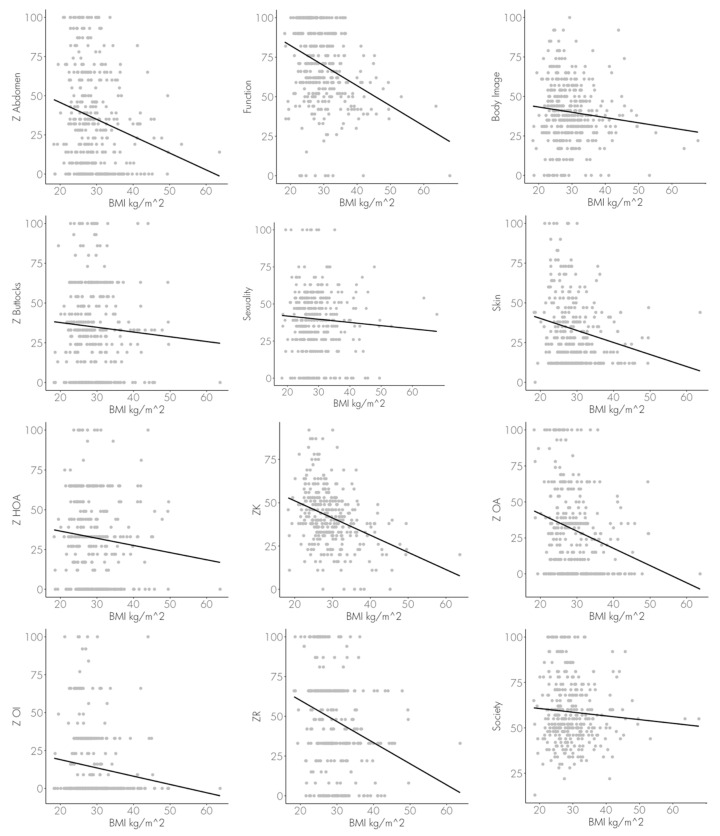
Regression analysis and Pearson’s correlation of the patients’ BMI and selected headings of the BODY-Q questionnaire: body image, sexuality, society, satisfaction-abdomen (Z Abdomen), satisfaction-body (ZK), satisfaction-upper arms (ZOA), satisfaction-back (ZR), satisfaction-buttocks (Z Buttock), satisfaction-hips and outer thighs (ZHOA), satisfaction-inner thighs (ZOI), assessment of excess skin (Skin).

A total of 355 patients (77.17%) presented with a BMI ≥ 25 kg/m^2^. The mean BMI was 29.54 kg/m^2^ with an interquartile range from 25.295 kg/m^2^ to 32.374 kg/m^2^. A significant relationship between high BMI and low QoL was observed in the following scales: body function, ZOA, ZR, ZK, ZOI and Skin. *p*-values, Pearson’s correlation coefficient (R) and its coefficient of determination (R^2^) are listed in Table 3. Figure 2 shows the distribution of the respective values of the BODY-Q subscales as a function of BMI.

Based on the amount of weight loss, participants were divided into four groups. Group A (n = 79; 16.5%) reported weight loss from 0 kg to 30 kg, Group B (n = 123; 26.7%) from 30 kg to 50 kg, Group C (n = 145; 31.5%) from 50 kg to 70 kg and Group D (n = 113; 25%) more than 70 kg. Table 4 lists the significant subcategories including *p*-values and Chi^2^-values.

**Figure 3:**
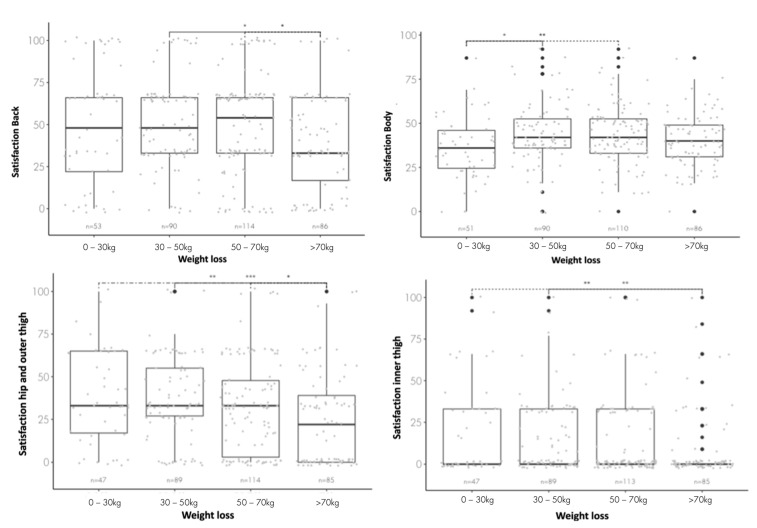
Representation of satisfaction-back/-body/-hip and outer thigh/-inner thigh categorized according to the amounts of weight lost. The boxplots show the median with the corresponding interquartile range and the distribution of the group. The statistical differences were calculated using the Kruskal–Wallis test and the subsequent post-hoc analysis was performed using the pairwise Wilcoxon test; significance level: * *p* < 0.1; ** *p* < 0.05, *** *p* < 0.01.

In the subcategory ZR, significant differences were found between B vs. D (*p* = 0.062 *) and C vs. D (*p* = 0.062 *) (Figure 3).

In the subcategory ZK, significant differences were found between group A vs. B (*p* = 0.055 *) and A vs. C (*p* = 0.042 *) (Figure 3).

In the subcategory ZHOA, significant differences were found between group A vs. D (*p* = 0.0239), B vs. D (*p* = 0.0039) and C vs. D (*p* = 0.0569) (Figure 3).

In the subcategory ZOI, significant differences were found between group A vs. D (*p* = 0.046 *) and B vs. D (*p* = 0.013 *) (Figure 3).

## 4. Discussion

Globally, the burden of obesity and its comorbidities is significantly increasing [24]. Over the past 30 years, a steady growth of obesity prevalence has been observed in OECD countries [25]. In fact, obesity mostly results in respiratory and cardiovascular comorbidities, going along with an increased risk of mortality [26,27,28]. Not only physical, but also psychological well-being can be significantly diminished by obesity. Discomfort and insecurity might lead to a decrease in self-confidence, psychosocial interaction and QoL [14]. So far, literature only partially reports on the impact/effectiveness of weight reduction as a method for health-related quality of life (HRQoL) improvement [4,13,14].

The body mass index (BMI) is now considered as an objectifiable, internationally standardized parameter for classifying body weight and also meets with general understanding and acceptance. The health status of a population can further be assessed using the BMI [4]. Based on the WHO classification [7], patients in our study can be currently classified as preadipose or obesity grade I (BMI = 29.54 kg/m^2^ (25.3–32.37 kg/m^2^) [29].

Kolotkin et al. studied the change in HRQoL of obese patients after at least one year of conservative therapy [30] using the questionnaire “The Impact of Weight on Quality of Life-Lite (IWQoL-Lite)” [31]. The participants were able to reduce their weight by 17.6% on average. A linear relationship as well as a significant positive change in physical functions, self-esteem and sexual life were evident indicating a correlation between weight reduction and HRQOL [30].

Petersen et al. further reported a negative correlation between obesity and HRQoL [32]. Thereby, QoL was measured using the Short Form 36 (SF36) questionnaire. As in the study conducted, the cohort was divided based on the BMI. Regression analyses of the study cohort examined, statistically confirmed that HRQoL is weakened by a high BMI. This is strongly supported by our results. Conclusively, Petersen et al. came to the conclusion that a high weight limits QoL on a physical and psychological level [32].

Likewise, in our study, a significant association between weight and QoL was found (*p* < 0.05) in the categories assessing body image, body function, skin assessment, and ZOA, ZR, ZK and ZOI. The higher the BMI, the lower the health related QoL of the patients. Furthermore, the same trend can be seen regarding sexuality, society, Z-Abdomen, Z-Buttocks and ZHOA, clearly demonstrating that a healthy body weight is indispensable for a high QoL. Therefore, guidance for long term weight reduction should be the primary treatment goal with obese patients. According to the literature, a 10–20% reduction is sufficient for a significant increase in QoL [33].

Regarding the method of weight loss, Kolotkin et al. showed an improvement in psychological and physical aspects of HRQoL after weight reduction [30]. The authors stated that there is a correlation between weight loss after bariatric surgery and improvement in HRQoL. Due to the fact, this was only found in some patients, they stated that HRQoL should be investigated in more detail [30].

When comparing conservative to surgical weight loss, no difference in QoL could be detected. These findings are supported by the results of Bischof et al., using the SF36 [34] confirming a significant improvement in the HRQoL of massively overweight patients by weight reduction. In addition, they also concluded that the way of weight loss is not decisive.

In order to be able to determine the weight loss and it’s change in HRQoL quantitively, the cohort was subdivided based on weight loss. Multiple significant differences were found between the weight loss classes-noticeably focused on the trunk as well as the lower part of the body. The rubric ZHOA as well as ZOI shows a linear decrease in quality of life from the lowest weight loss (0–30 kg) to the highest weight loss (>70 kg). However, it should be noted that the rubric ZOI has the lowest scores. The low HRQoL despite a large weight reduction could be due to dissatisfaction caused by massive skin overhangs and thereby caused mechanical and aesthetic obstacles.

In contrast to the lower extremities, the back is not affected by a weight loss of more than 70 kg, with an average HRQoL of 39.38. In the remaining three weight classes, almost identical scores of around 50.17 HRQoL can be found. While the dorsal area (back) has a significantly firmer skin, the subcutaneous fat tissue of the back is more prominent [35] compared with the ventral part of the body [36,37,38]. Additionally, focus is on the front side of the body. All these aspects may be reasons why there is no significant difference between the weight categories in the category of ZR.

Nevertheless, it can be reported that the satisfaction with the body in general is significantly higher with a weight reduction of 30–50 kg and 50–70 kg than with a reduction of 0-30 kg. Hence supporting previous studies, we clearly showed that any kind of weight loss has a positive effect on QoL—the more weight is lost, the higher the effect is.

## 5. Conclusions

Obesity and being overweight can have a significant impact on the quality of life. A high BMI correlates negatively with body image and individual assessment of most body regions. Increased weight loss is associated with significant improvement of quality of life and can thus counteract the reduction in quality of life experienced previously. The method of weight loss (conservative vs. surgical) does not influence the QoL. It is remarkable, that in our analysis, patients with a weight loss between 30 and 70 kg showed the highest general-body satisfaction.

### Limitations

As a cross-sectional study, our analysis only provides a snapshot of the cohort at the time of the online survey. Similar to other questionnaire-based studies, only a small proportion of participants in the cohort studied were male [30,39]. German language skills as well as an internet-enabled device were required for participation.

The different group sizes in the subcategories can be considered as a further limitation. Only complete response of a subgroup led to inclusion in the subgroup analysis. This is due to a drop-out rate caused by the 164 questions of the Body Q. Thereby, a decreasing trend in completeness and number of participants with increasing length of the BODY-Q was observed. Additionally, comparable to other questionnaire-based studies, men represented only the minority of respondents (3.7%)—therefore no gender subgroup analysis has been performed. Correlation and comparison with previous studies remains difficult due to the inhomogeneity of the questionnaire type, inclusion criteria and patient size.

## Figures and Tables

**Table 1:** Classification of weight classes based on the WHO scheme [7].

BMI	Weight Classes
<18.5 kg/m^2^	Under weight
18.5–24.9 kg/m^2^	Normal weight
25.0–29.9 kg/m^2^	Pre-obesity (overweight)
30.0–34.9 kg/m^2^	Obesity grade I
35.0–39.9 kg/m^2^	Obesity grade II
≥40 kg/m^2^	Obesity grade III

**Table 2:** Presentation of the mean BODY-Q values in the categories psychosocial, body image, function, sexuality, society and skin, satisfaction-abdomen (Z Abdomen), satisfaction-upper arms (ZOA), satisfaction-back (ZR), satisfaction-body (ZK), satisfaction-buttocks (Z Buttock), satisfaction-hips and outer thighs (ZHOA), satisfaction-inner thighs (ZOI) for the groups weight reduction by means of conservative and surgical measures, with standard deviation (SD) in brackets.

	Psychosocial	Body Image	Function	Sexuality	Society	Skin	
**conservative**	51.58 (±1.84)	37.86 (±2.10)	73.37 (±2.42)	42.04 (±1.97)	58.17 (±1.90)	33.71 (±3.00)	
**surgical**	43.29 (±1.15)	40.85 (±1.03)	69.33 (±1.41)	39.09 (±1.27)	58.88 (±1.06)	32.92 (±1.35)	
						
	**Z Abdomen**	**ZOA**	**ZR**	**ZK**	**Z Buttock**	**ZHOA**	**ZOI**
**conservative**	33.56 (±3.47)	34.36 (±3.12)	51.18 (±3.08)	39.90 (±1.91)	34.72 (±3.01)	35.53 (±3.14)	17.31 (±3.01)
**surgical**	36.14 (±1.88)	29.19 (±1.83)	46.39 (±1.89)	42.42 (±1.02)	34.95 (±1.66)	31.43 (±1.65)	12.85 (±1.41)

**Table 3:** Presentation of the correlation coefficients according to Pearson (r) for the categories body image, function, sexuality, society, skin, satisfaction-stomach (ZAbdomen), satisfaction-upper arms (ZOA), satisfaction-back (ZR), satisfaction-body (ZK), satisfaction-buttocks (ZButtocks), satisfaction-hips and outer thighs (ZHOA), satisfaction-inner thighs (ZOI), of the BODY-Q questionnaire and the patients’ BMI; the *p*-values; the coefficient of determination (R^2^); * A correlation level of 0.05 was assumed to be significant.

	Body Image	Function	Sexuality	Society		Skin	
***p*-value**	0.023 *	<0.001 *	0.203	0.166		<0.001 *	
**r**	−0.113	−0.335	−0.066	−0.073		−0.203	
**R^2^**	0.010	0.110	0.002	0.003		0.038	
					
	**Z Abdomen**	**ZOA**	**ZR**	**ZK**	**Z Buttock**	**ZHOA**	**ZOI**
***p*-value**	0.074	0.005 *	0.001 *	<0.001 *	0.221	0.066	0.011 *
**r**	−0.209	−0.242	−0.264	−0.357	−0.066	−0.101	−0.139
**R^2^**	0.041	0.056	0.067	0.125	0.001	0.007	0.016

**Table 4:** Representation of the *p*-values for the categories satisfaction-back (ZR), satisfaction-body (ZK), satisfaction-the hips and outer thighs (ZHOA), satisfaction-inner thighs (ZOI), of the BODY-Q questionnaire and according to the breakdown into the weight loss groups; * The correlation is significant at the 0.05 level.

	ZR	ZK	ZHOA	ZOI
***p*-value**	0.040 *	0.019 *	0.003 *	0.013 *
**Chi-square**	8312	99,565	13,975	10,742

## Data Availability

Not applicable.

## References

[1] Schienkiewitz A., Mensink G., Kuhnert R., Lange C. (2017). Übergewicht und Adipositas bei Erwachsenen in Deutschlan. J. Health Monit..

[2] Biraima-Steinemann M., Maurer S., Angst E. (2016). Konservative Therapie der Adipositas. Ther. Umsch..

[3] González-Muniesa P., Mártinez-González M.A., Hu F.B., Després J.-P., Matsuzawa Y., Loos R.J.F., Moreno L.A., Bray G.A., Martinez J.A. (2017). Obesity. Nat. Rev. Dis. Prim..

[4] Pyschkin A. (2020). Abdominoplastik Nach Massivem Gewichtsverlust. Eine Analyse von Komplikationen und der Entwicklung Postoperativer Lebensqualität. Ph.D. Thesis.

[5] Hauner H., Buchholz G., Hamann A., Husemann B., Koletzko B., Liebermeister H., Wabitsch M., Westenhöfer J., Wirth A., Wolfram G. (2007). Prävention und Therapie der Adipositas. Evidenzbasierte Leitlin. Version.

[6] Weir C.B., Jan A. (2019). BMI Classification Percentile and Cut Off Points.

[7] WHO Body Mass Index-BMI. https://www.euro.who.int/en/health-topics/disease-prevention/nutrition/a-healthy-lifestyle/body-mass-index-bmi.

[8] Hauner H. (2018). Prävention und Therapie der Adipositas. Der Junge Zahnarzt.

[9] (2016). BDDNN. Damit Jetzige und Kommende Generationen Gut Leben Können.

[10] Böcken J., Braun B., Landmann J. (2010). Gesundheitsmonitor 2009: Gesundheitsversorgung und Gestaltungsoptionen aus der Perspektive der Bevölkerung.

[11] Umbach G. (2014). Kosten-Nutzen-Bewertungen. Erfolgreich als Medical Advisor und Medical Science Liaison Manager.

[12] Schneider F., Wien S., Weber-Papen S. (2017). Epidemiologie und Ätiologie psychischer Erkrankungen. Facharztwissen Psychiatrie, Psychosomatik und Psychotherapie.

[13] Meier A.C. (2020). Lebensqualität und Zufriedenheit Nach Ästhetischer Abdominoplastik–eine Prospektive Studie. PhD Thesis.

[14] Kolotkin R.L., Andersen J.R. (2017). A Systematic Review of Reviews: Exploring the Relationship Between Obesity, Weight Loss and Health-Related Quality of Life. Clin. Obes..

[15] Bevans M. (2010). Health-Related Quality of Life Following Allogeneic Hematopoietic Stem Cell Transplantation. Hematol. Am. Soc. Hematol. Educ. Program.

[16] Karimi M., Brazier J. (2016). Health, Health-Related Quality of Life, and Quality of Life: What is the Difference?. Pharmacoeconomics.

[17] de Ligt K.M., de Rooji B.H., Hedayati E., Karsten M.M., Smaardijk V.R., Velting M., Saunders C., Travado L., Cardoso F., Lopez E. (2023). International Development of a Patient-Centered Core Outcome Set for Assessing Health-Related Quality of Life in Metastatic Breast Cancer Patients. Breast Cancer Res. Treat..

[18] Bullinger M. (2016). Zur Messbarkeit von Lebensqualität. Lebensqualität in der Medizin.

[19] Sarwer D.B., Lavery M., Spitzer J.C. (2012). A Review of the Relationships Between Extreme Obesity, Quality of Life, and Sexual Function. Obes. Surg..

[20] Sarwer D.B., Fabricatore A.N. (2008). Psychiatric Considerations of the Massive Weight Loss Patient. Clin. Plast. Surg..

[21] Strasser B., Pichler B. (2004). Diet and Physical Activity in the Treatment of Obesity. Wien Med. Wochenschr..

[22] Klassen A.F., Kaur M., Breitkopf T., Thoma A., Cano S., Pusic A. (2018). Using the BODY-Q to Understand Impact of Weight Loss, Excess Skin, and the Need for Body Contouring following Bariatric Surgery. Plast. Reconstr. Surg..

[23] Klassen A.F., Cano S.J., Alderman A., Soldin M., Thoma A., Robson S., Kaur M., Papas A., Van Laeken N., Pusic A.L. (2016). The BODY-Q: A Patient-Reported Outcome Instrument for Weight Loss and Body Contouring Treatments. Plast. Reconstr. Surg. Glob. Open.

[24] Tönsmeyer T., Wieters H. (2022). Welt–Hunger–Hilfe. Zur Zeitgeschichte eines Menschheitsproblems. Zeithistorische Forsch. –Stud. Contemp. Hist..

[25] OECD (2017). Obesity Update 2017.

[26] Adams K.F., Schatzkin A., Harris T.B., Kipnis V., Mouw T., Ballard-Barbash R., Hollenbeck A., Leitzmann M.F. (2006). Overweight, Obesity, and Mortality in a Large Prospective Cohort of Persons 50 to 71 Years Old. N. Engl. J. Med..

[27] Kurth T., Gaziano J.M., Berger K., Kase C.S., Rexrode K.M., Cook N.R., Buring J.E., Manson J.E. (2002). Body Mass Index and the Risk of Stroke in Men. Arch. Intern. Med..

[28] Zammit C., Liddicoat H., Moonsie I., Makker H. (2010). Obesity and Respiratory Diseases. Int. J. Gen. Med..

[29] Mensink G.B.M., Schienkiewitz A., Haftenberger M., Lampert T., Ziese T., Scheidt-Nave C. (2013). Übergewicht und Adipositas in Deutschland. Bundesgesundheitsblatt-Gesundh. -Gesundh..

[30] Kolotkin R.L., Crosby R.D., Williams G.R., Hartley G.G., Nicol S. (2001). The Relationship Between Health-Related Quality of Life and Weight Loss. Obes. Res..

[31] Quality Of Life Consulting IWQOL-Lite. https://www.qualityoflifeconsulting.com/iwqol-lite.html.

[32] Petersen I., Burgmer R., Herpertz S. (2007). Adipositas und Lebensqualität–ein Kontrollierter Vergleich Normal-und Übergewichtiger Stichproben. PPmP-Psychother. Psychosom.·Med. Psychol..

[33] Sammet I., Dammann G., Wiesli P., Müller M. (2016). Adipositas: Interdisziplinäre Behandlung und psychosomatische Perspektive.

[34] Bischoff G., Imaguire C., Heidenreich T., Tschochner R., Hagen H., Wechsler J.G. (2004). Einfluss einer Gewichtsreduktion auf die Gesundheitsbezogene Lebensqualität (SF 36). Aktuelle Ernährungsmedizin.

[35] Baranova A., Collantes R., Gowder S.J., Elariny H., Sclauch K., Younoszai A., King S., Randhawa M., Pusulury S., Alsheddi T. (2005). Obesity-Related Differential Gene Expression in the Visceral Adipose Tissue. Obes. Surg..

[36] Richter D.F., Stoff A., Velasco-Laguardia F.J., Reichenberger M.A. (2008). Circumferential Lower Truncal Dermatolipectomy. Clin. Plast. Surg..

[37] Hönig J.F. (2008). Anatomie und Physiologie der Abdominalwand. Abdominoplastik: Prinzip und Technik.

[38] Klöting N., Stumvoll M., Blüher M. (2007). Biologie des Viszeralen Fetts. Der Internist.

[39] Bragg T.W., Jose R.M., Srivastava S. (2007). Patient Satisfaction Following Abdominoplasty: An NHS Experience. J. Plast. Reconstr. Aesthet. Surg..

